# Evidence for the stability of the West Antarctic Ice Sheet divide for 1.4 million years

**DOI:** 10.1038/ncomms10325

**Published:** 2016-02-03

**Authors:** Andrew S. Hein, John Woodward, Shasta M. Marrero, Stuart A. Dunning, Eric J. Steig, Stewart P. H. T. Freeman, Finlay M. Stuart, Kate Winter, Matthew J. Westoby, David E. Sugden

**Affiliations:** 1 School of GeoSciences, University of Edinburgh, Drummond Street, Edinburgh EH8 9XP, UK; 2 Department of Geography, Northumbria University, Ellison Place, Newcastle upon Tyne NE1 8ST, UK; 3 Department of Geography, School of Geography, Politics and Sociology, Newcastle University, Newcastle upon Tyne NE1 7RU, UK; 4 Quaternary Research Center and Department of Earth and Space Sciences, University of Washington, Seattle, Washington 98195, USA; 5 Scottish Universities Environmental Research Centre, Rankine Avenue, East Kilbride G75 0QF, UK

## Abstract

Past fluctuations of the West Antarctic Ice Sheet (WAIS) are of fundamental interest because of the possibility of WAIS collapse in the future and a consequent rise in global sea level. However, the configuration and stability of the ice sheet during past interglacial periods remains uncertain. Here we present geomorphological evidence and multiple cosmogenic nuclide data from the southern Ellsworth Mountains to suggest that the divide of the WAIS has fluctuated only modestly in location and thickness for at least the last 1.4 million years. Fluctuations during glacial–interglacial cycles appear superimposed on a long-term trajectory of ice-surface lowering relative to the mountains. This implies that as a minimum, a regional ice sheet centred on the Ellsworth-Whitmore uplands may have survived Pleistocene warm periods. If so, it constrains the WAIS contribution to global sea level rise during interglacials to about 3.3 m above present.

## Abstract (web summary)

Predicting the West Antarctic Ice Sheet's response to future warming is hindered by a lack of historical evidence. Here, based on geomorphological evidence from and cosmogenic dating of Ellsworth Mountains' deposits, the authors show that at least a regional ice sheet survived Pleistocene interglacial cycles.

## Graphical abstract

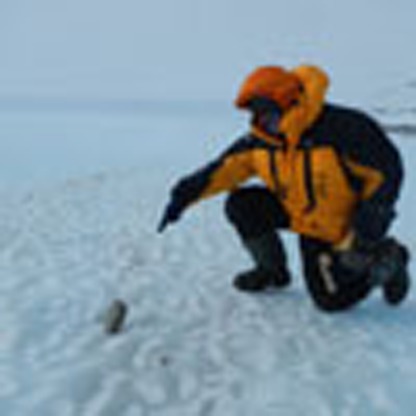

The West Antarctic Ice Sheet (WAIS) is pinned on an archipelago with its central dome situated over subglacial uplands and bedrock basins, the latter more than 1,500 m below sea level (Fig. 1). For over four decades there has been a fear that this topography could lead to marine instability, since retreat of the ice margin into the basins would enhance ice calving and ice-mass loss, leading to loss of the WAIS and a rapid rise in global sea level of 3–5 m (refs 1, 2, 3). Recent studies have suggested that such a collapse may already be underway in the Pacific-facing sector of the ice sheet^4^,^5^. Constraining the past ice behaviour would allow a more confident assessment of its potential contribution to past and future sea level change. Marine biological evidence based on diatoms and the similarity of octopus and *Bryozoa* between the Pacific and Atlantic sectors suggests that much of the ice sheet disappeared during interglacials, creating an open seaway between these sectors^6^,^7^,^8^. Such a conclusion is reinforced by estimates of higher-than-present global sea levels during interglacials^9^,^10^. Efforts to constrain the minimum configuration of the ice sheet in the past have relied on numerical ice-sheet models, each with its own set of assumptions on boundary conditions, internal dynamics and external forcing (for example, climate and sea level)^3^,^11^,^12^. The models suggest that most upland areas could have remained glaciated even during the warmest interglacials, but whether as individual mountain glaciers or as larger regional ice sheets remains uncertain, and there is no direct evidence from the continent to constrain this.

The Heritage Range, situated in the heart of the Weddell Sea embayment, lies within 50 km of the interface between the grounded ice sheet and the floating Filchner-Ronne Ice Shelf in Hercules Inlet (Fig. 1). Two component massifs, Patriot Hills and Marble Hills are summits of a 15-km-wide upland bounded by troughs excavated to below sea level^13^. At present, ice from the central WAIS flows around and between these mountains to the grounding line. The WAIS divide forms a broad saddle between the main dome 300 km to the west and another 200 km to the northwest. Katabatic winds flow down the ice slope from the divide towards Hercules Inlet crossing the mountains and creating blue-ice areas in their lee (Fig. 1). The winds cause ablation of surface ice that in turn causes a compensating upward flow of ice that brings basal debris to the ice-surface as blue-ice moraines^14^,^15^. This ice-marginal, basally derived material is deposited higher on the mountain flanks and records past changes in ice thickness.

The use of cosmogenic nuclide dating on bedrock and glacially transported material on nunataks in Antarctica has provided much quantitative data on the history of ice thickness changes over time^16^,^17^,^18^,^19^,^20^,^21^,^22^,^23^. Initial work in the Heritage Range revealed a scatter of ages of up to 400 ka in elevated blue-ice moraines^24^,^25^. These data led to the untested hypothesis that the spread in ages represented the continuous presence of an ice sheet that fluctuated in thickness in response to glacial–interglacial cycles^14^. The range of ages reflects preservation of some erratics and deposition of others during successive glaciations. An alternative possibility is that the moraines represent composite features formed by multiple ice-sheet inundations interspersed with periods of local mountain glaciation or deglaciation.

The combination of geomorphological analysis of landforms and measurement of multiple cosmogenic nuclides can provide rare insight into ice-sheet history. The advantage of measuring multiple cosmogenic nuclides in single samples is that both the age and exposure history can be constrained^22^. For example, if a previously exposed clast is buried by ice long enough for the shorter lived of two nuclides to decay preferentially, the signal will be observed in the isotopic ratio^26^. In the case of cosmogenic ^26^Al/^10^Be, it takes several tens of thousands of years for the burial signal to become evident. By measuring multiple isotopes in three adjacent erratics at each specific sampling site, the degree of scatter and extent to which the erratics have shared the same history of exposure can be determined. Thus, one can gain information on the age of deposition and possible subsequent overriding and disturbance by ice.

Here we use geomorphological analysis of landforms and deposits supported by *in situ* cosmogenic ^26^Al, ^10^Be and ^21^Ne from newly collected, quartz-bearing erratics to investigate elevated blue-ice moraines. Our evidence reveals several relict ice-marginal blue-ice moraine deposits as old as 1.4 Ma. The isotopic evidence indicates that the highest deposits have not been disturbed by ice since deposition, but lower deposits have experienced subsequent burial. All geomorphic and cosmogenic nuclide data are consistent with an ice sheet that thickened and thinned in response to quaternary glacial–interglacial cycles. We find no evidence to suggest a change in glaciological conditions that would accompany the loss of the entire ice sheet and the build-up of individual mountain glaciers. We interpret this consistency as evidence for continuous ice-sheet conditions in this part of the Weddell Sea sector. The minimum configuration that maintains strong katabatic winds is a regional ice sheet centred on the Ellsworth-Whitmore block. This interpretation, where the WAIS shows dynamic equilibrium about a continuous ice divide, supports numerical models that indicate a maximum WAIS contribution to sea level of about 3.3 m (refs 3, 11), consistent with low-end estimates of global sea level during past interglacial periods^9^. Such an interpretation is also consistent with marine biological evidence indicating an open seaway in West Antarctica during some interglacials^6^,^7^,^8^.

## Results

### Geomorphology

**Figure 1:**
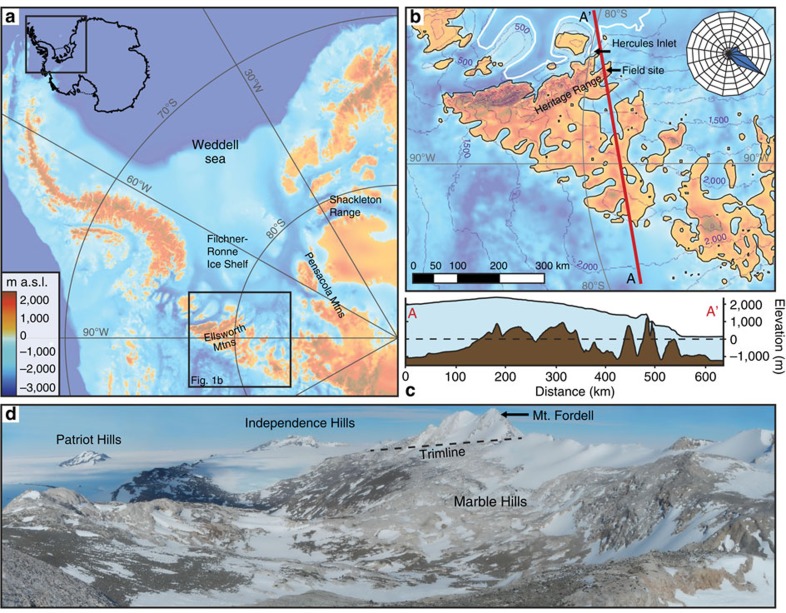
The Heritage Range field site. (**a**) Subglacial topography of Antarctica^57^ showing the field location in the Ellsworth Mountains and prominent geographical features within the Weddell Sea embayment; inset shows wider Antarctic setting. (**b**) Subglacial topography of the wider Ellsworth block and the present-day ice-surface contours (250 m) of the WAIS. White line indicates the grounding line^58^; black lines are bed elevation contours at 0 m elevation (WGS84). The red line A-A' shows the profile line used in **c**; it runs between the main dome of the WAIS and Hercules inlet. The rose diagram shows persistent katabatic winds from the south-southwest recorded at the Patriot Hills blue-ice aircraft runway over two months in the austral summer of 2008 (ref. 59). (**c**) Profile of the bed and ice-sheet surface from the WAIS divide to Hercules Inlet, showing the deep troughs excavated below sea level surrounding the Patriot and Marble Hills. (**d**) Photograph showing dark-coloured erratics scattered across ice-scoured limestone bedrock of the Marble Hills. Wind-drift glaciers can be seen along the summit ridge to the right of Mt. Fordell (see Supplementary Fig. 2 for a geomorphic map).

**Figure 2:**
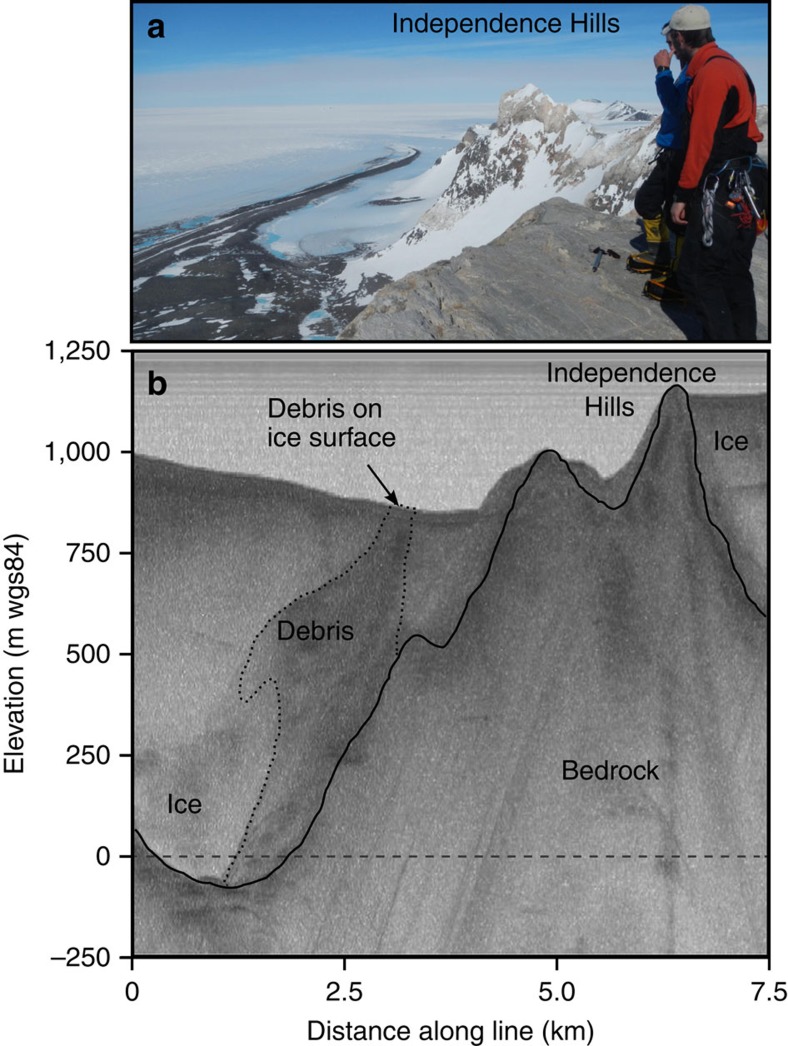
The source of blue-ice moraine debris. (**a**) The blue ice moraine at the foot of Independence Hills. (**b**) RES transect across blue-ice moraines adjacent to Independence Hills showing how debris underlies the folded surface moraines. The origin of the debris (dotted line), much of it locally derived, is from deep within the glacier trough, indeed close to present sea level. In addition to the lateral flow there is a limited longitudinal component of flow to the east (into the page). The 150 MHz pulse-processed radar data was collected by the British Antarctic Survey Polarimetric-radar Airborne Science Instrument ice-sounding radar in 2010/2011.

The geomorphological analysis of landforms and deposits reveals currently active blue-ice moraines at the edge of glaciers at the eastern foot of the mountains (Supplementary Figs 1–4). Striated, basally derived clasts occur in the moraines and in folded debris bands in the adjacent glacier surface. Airborne radio echo sounding (RES) data reveals that the debris sequences originate ∼800 m below at the glacier base at a depth close to present sea level (Fig. 2). Above the ice margin are two formerly glaciated zones marked by an upper erosional trimline (Fig. 1d), the latter recognized throughout the Ellsworth Mountains^27^. The upper weathered zone occurs up to 650 m above the present ice-surface and is covered by iron-stained, quartz-rich erratics and till patches on an ice-eroded limestone or marble bedrock surface. Lower down in this zone, the weathered erratics and till have been disturbed by eastward flowing ice. This is demonstrated by erratics preferentially trapped in irregularities in the bedrock and the preservation of till patches in basins and on the eastern side of bedrock bumps, leaving the western slopes and summits relatively free of debris. The weathered deposits represent former ice-marginal blue-ice moraines. This conclusion is borne out by the location and concentration of till patches at the foot of a mountain escarpment athwart katabatic winds, their proximity to a former ice margin and the lack of erratics above the trimline (Supplementary Fig. 4). Moreover, the shape and lithology of the erratics is the same as the quartz-rich lithologies in the moraines at the current ice edge. The lower unweathered zone is characterized by fresh erratics, perched boulders and ice-cored tills; it is thought to reflect deposition by ice during the Last Glacial Maximum^24^ and is not considered here.

### Cosmogenic nuclide data

**Figure 3:**
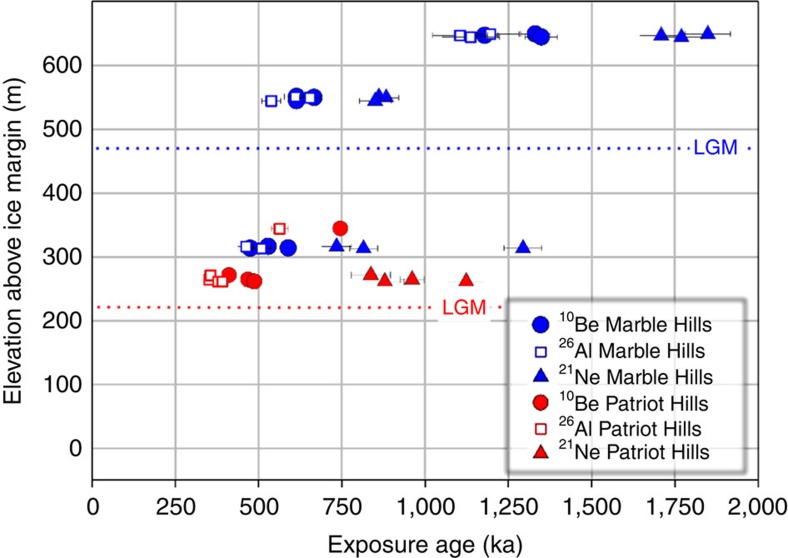
The cosmogenic nuclide data. The apparent exposure ages of weathered rock samples from the Marble Hills (blue) and Patriot Hills (red) plotted against elevation above the ice margin. Error bars (1σ) reflect analytical uncertainties only. There is a clear decline in exposure ages with decreasing elevation at the Marble Hills. Three Marble Hills samples with ^10^Be ages of 500–600 ka survived beneath the ice cover of the Last Glacial Maximum^24^ (blue dotted line). A similar exposure history involving burial is inferred for samples above the Last Glacial Maximum limit^24^ in the Patriot Hills (Supplementary Figs 5-6).

We measured *in situ* cosmogenic ^26^Al, ^10^Be and ^21^Ne on newly collected quartz-bearing erratics (see Methods and Supplementary Tables 1–3). The exposure ages of the weathered erratics decline with decreasing elevation towards the glacier surface (Fig. 3). Three adjacent erratics from each of two sites in the upper weathered zone in the Marble Hills have ^10^Be exposure ages of 1.2–1.4 Ma, and at a slightly lower elevation above the ice, 0.6–0.7 Ma. The samples at both sites yield tightly clustered ages for each isotope with ratios that do not indicate prolonged burial (see Methods and Supplementary Fig. 5). Lower down, erratics have younger ^10^Be exposure ages of 0.5–0.6 Ma with some isotopic evidence of burial. Comparable erratics from a patch of weathered till in the Patriot Hills have similarly clustered ^10^Be exposure ages of 0.4–0.5 Ma and isotopic ratios indicating more than 300 ka burial (Supplementary Fig. 6). Seven samples emerging from the ice today in front of both the Marble and Patriot Hills have exposure ages of <1.5 ka and thus total inheritance is low. The striking feature of the data from the high elevation samples is that they reflect a shared origin and exposure history. Rather than the scatter of ages one might expect with repeated episodes of burial by ice, there is a consistent pattern of decreasing age of exposure and increasing degree of burial towards the present glacier margin. This suggests that any subsequent burial at the different sites was by cold-based ice that did not move existing material or deposit new material in the process.

## Discussion

**Figure 4:**
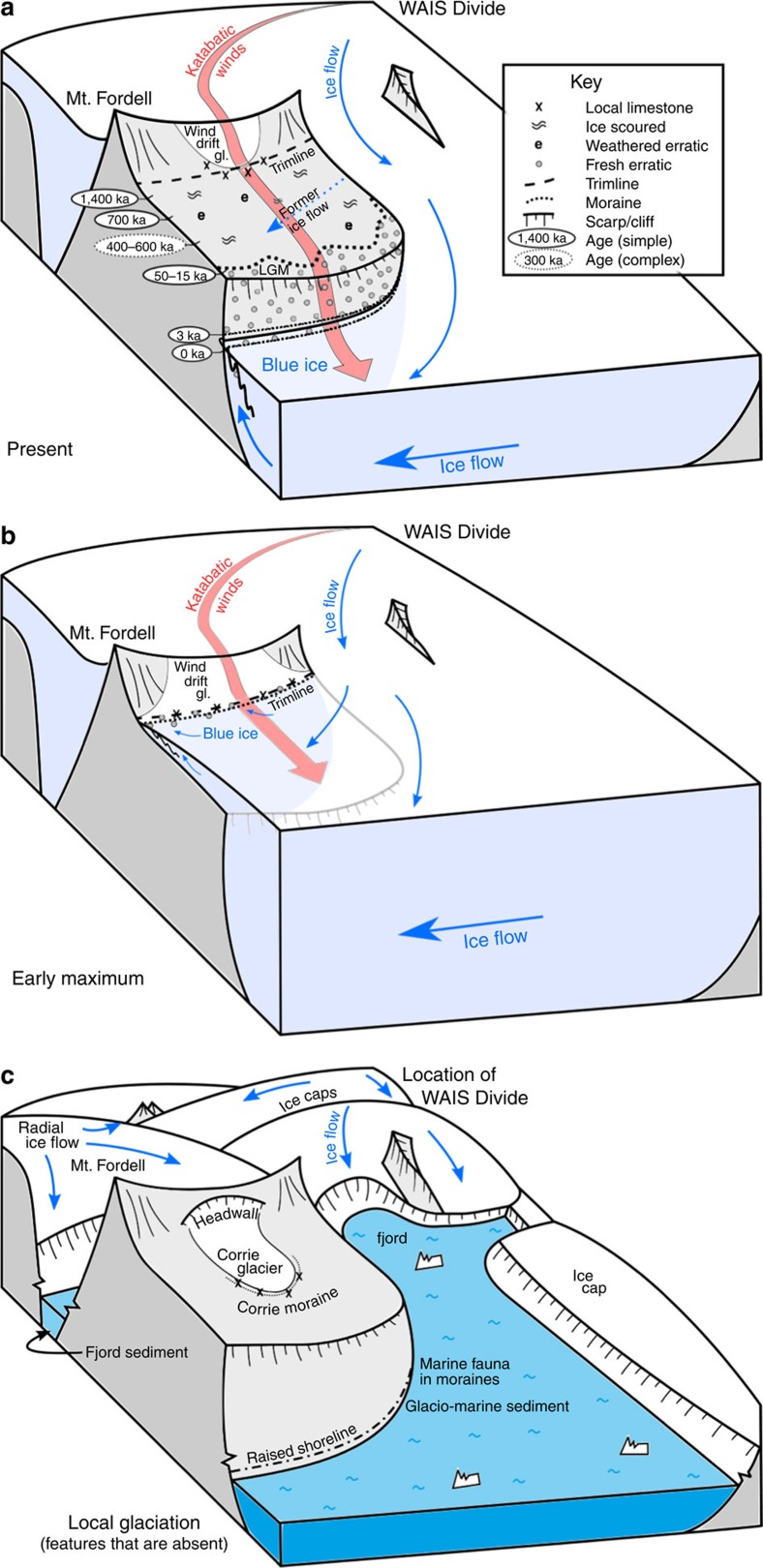
A conceptual reconstruction of different ice-sheet configurations surrounding the Marble Hills massif. The reconstruction (looking west) shows (**a**) the present minimum, (**b**) past maximum blue-ice relationships and (**c**) features that would accompany local glaciation. The ages of weathered quartz-rich erratics on the ice-scoured bedrock upland decline and their burial history becomes more complex at lower elevations, the latter reflecting longer periods of burial by ice. The Last Glacial Maximum limit is often the lowest on the upland plateau. The evidence is consistent with oscillations in ice thickness related to Pleistocene sea level fluctuations. There is no evidence of local glaciation radiating from the massifs.

The simplest explanation of the pattern of cosmogenic nuclide data is that an ice margin fluctuated in elevation on the mountain flank (Fig. 4). The highest erratics are exposed for the longest time, while progressively lower erratics are exposed for increasingly shorter periods of time. This explains both the younging trend and evidence of increased burial with decreased altitude. The implication of exposure ages of up to 1.4 Ma at higher elevations is that ice thickening and blue-ice moraine formation also occurred during earlier glacial cycles in the Pleistocene. Given the mountains are situated near the grounding line of today, increases in ice thickness near the mountains would accompany any seaward migration of the grounding line as ocean temperature cooled and global sea level fell^28^. Over millions of years one would expect glacial erosion to lower the ice-sheet surface relative to the mountains^29^,^30^ and thus the cyclic changes in ice thickness would be superimposed on a trajectory of lowering relative to the mountains. This scenario is consistent with the great ages and minimal burial of the highest erratics.

It could be argued that the scenario above should produce a scatter of exposure ages of up to 1.4 Ma, rather than clustering at certain ages. Indeed, such scatter has been measured in tills within the lower unweathered zone in the Marble Hills, which ranged from 15 to 250 ka (ref. 24). The clustering may reflect episodes in the past when conditions were particularly favourable for the formation of blue-ice moraines at those particular locations, just as the modern concentration of blue-ice material varies with local topography and ice-surface elevation. In addition, clustering would be an expected artefact of the sampling, which was concentrated on sites at specific altitudes.

The implication of the evidence above is that the WAIS divide and associated katabatic winds have also been present for at least 1.4 Ma. The blue-ice moraines form because of strong katabatic winds and these in turn are strongest and most consistent when they flow downslope for hundreds of km. The loss of the ice divide would diminish both katabatic winds and blue-ice moraine formation.

Could such an assemblage of deposits survive loss of the WAIS? The recorded ages leave adequate intervals of time for the ice divide to disappear. If the ice sheet disappeared, ice caps and glaciers would likely build up on mountain massifs in a fjord landscape. Each massif would have a different and locally radial pattern of flow depending on the type and scale of the topography (Fig. 4c). We found none of the features characteristic of such a scenario. Rather than radial flow from the mountain axis, Marble and the Patriot Hills bear geomorphologic evidence of eastward ice flow (Supplementary Fig. 4). In other fjord areas of the world, glacial deposits typically include marine traces, such as diatoms, shells and glaciomarine muds^31^. Examination of the Heritage Range tills, that RES shows are sourced from deep within the glacier troughs (Fig. 2), revealed no traces of marine diatoms or other biogenic silica in either present-day or elevated blue-ice tills (R. Scherer, personal communication, 2014). Local glaciation typically produces deposits associated with local corrie glaciers, as in the Asgard and Olympus ranges in the Transantarctic Mountains^32^. Rather than corrie glacier deposits, concentrations of material with a local origin in the study area are restricted to wind-drift glaciers that merged local rocks with exotic material in blue-ice moraines. Indeed, the very existence of former wind-drift glaciers supports the existence of the ice sheet and associated katabatic winds.

The lack of evidence of marine and local glaciation cannot on its own rule out short periods of complete deglaciation. The cosmogenic nuclide data alone are not a direct test of this hypothesis. It is possible that some evidence may remain preserved beneath the ice sheet or that the characteristic geomorphology is missing or poorly developed. Corrie and wind-drift glaciers could produce geomorphology that may be indistinguishable, while cold-based glaciers may leave no mark at all. Furthermore, interglacial periods are relatively short lived.

While we recognize the above possibility of complete deglaciation, there are arguments in favour of persistent glaciation. Recent atmospheric modelling of the Antarctic climate response to a collapse of the WAIS indicates significant warming would occur in the Atlantic sector of the WAIS^33^. Any such warming in an Antarctic maritime environment would cause an increase in snowfall and the growth of mountain glaciers. The re-glaciation of the WAIS would begin on upland areas such as the Heritage Range. Moreover, modelling suggests the increased temperature and accumulation relative to today would favour the formation of warm-based local glaciers that are efficient at removing sediment from their beds^12^. However, even if re-glaciation involved cold-based glaciers, which can move sediment selectively^32^,^34^, one would expect more scatter in the exposure age results, especially in the highest samples on Mt Fordell; these samples are situated high on the mountain and within 40 m of the present wind-drift glacier margin. Instead of scatter, the exposure ages are tightly clustered. In summary, while we acknowledge the limitations of our evidence, it seems significant to draw attention to the nature of the cosmogenic and geomorphological evidence from the inner reaches of the Weddell Sea embayment, which point to ice-sheet glaciation for 1.4 Ma.

**Figure 5:**
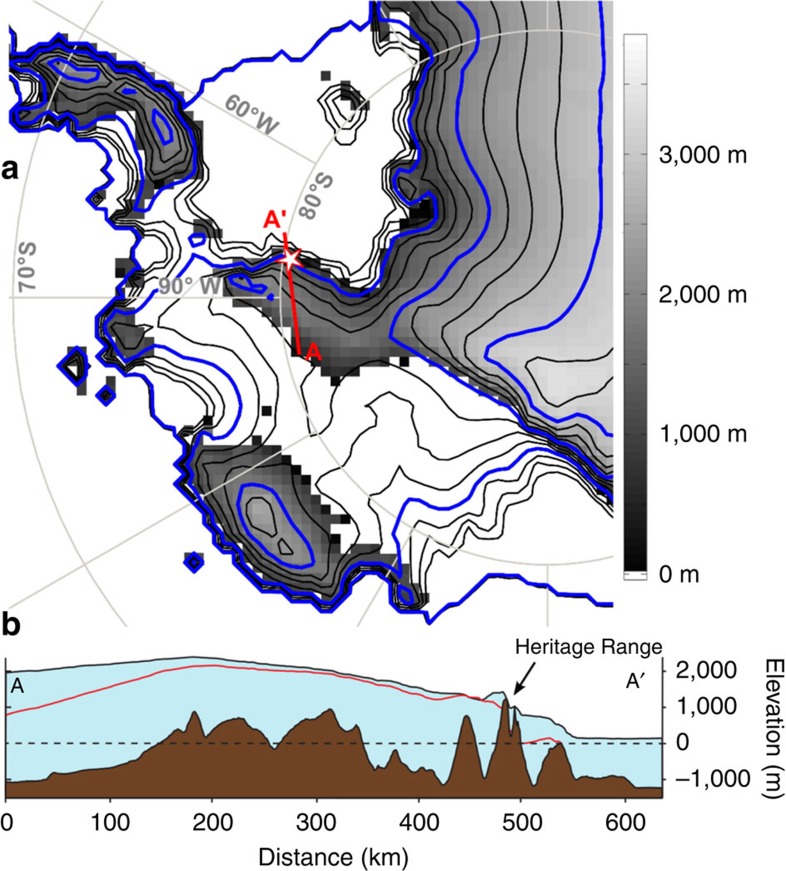
The minimum ice-sheet configuration. (**a**) Comparison of the modelled minimum Pleistocene extent of the WAIS^11^ in grey shading with the present ice-sheet surface represented by black contours (250 m); the blue contours indicate 1,000 m increments. The star indicates the Heritage Range. The catchment area for katabatic winds affecting the Heritage Range is comparable. The red line A-A' shows the profile line used in **b**; it runs between the main dome of the WAIS and Hercules inlet. (**b**) cross-section along A-A' showing the same present ice and bed topography^57^ used in Fig. 1c and the modelled minimum ice-sheet surface (red line)^11^. The regional ice-sheet surface remains at a similar elevation as the WAIS today. The latter surface was generated by taking the difference between the modelled present-day and minimum (205 ka) ice thickness and then subtracting this value from the present-day ice-sheet surface elevation. Small isostatic changes in bedrock elevation are accounted^11^ but are not shown.

One possible explanation for persistent ice-sheet conditions for at least 1.4 Ma is that the whole WAIS survived intact. Our evidence is compatible with this view, but the evidence for substantial increases in eustatic sea level during past interglacial periods^9^,^35^ and the evidence of marine connectivity between the Pacific and Atlantic marine sectors^6^,^7^,^8^ argues against the idea. Instead, we suggest our evidence points to the survival during interglacials of a smaller regional ice sheet centred on the upland of the Ellsworth-Whitworth massif to the south and west of the Ellsworth Mountains; an ice divide survived on the upland, while ice was lost from surrounding marine basins. Such a scenario during interglacials at 1.2 Ma and 205 ka has been simulated with an ice-sheet model, based on the assumption that ocean-driven melting at the ice-sheet margins is the primary determinant of changes in the WAIS^11^ (Fig. 5). The catchment area and fetch for katabatic winds crossing the Heritage Range remains approximately the same. Further, because the regional ice-sheet centres on a topographic high, the ice-surface altitude at the divide decreases by just a few hundred metres during interglacials and remains essentially the same in the vicinity of the Heritage Range (Fig. 5b). The preservation of a high WAIS divide would help explain the ∼7 Ma record of continuous high polar conditions in the adjacent Transantarctic Mountains, inferred from some of the lowest bedrock erosion rates in Antarctica^20^. This reconstruction also permits marine interaction between the Weddell Sea and the Amundsen Sea as suggested based on the analysis of diatoms, *Bryozoa* and octopus^6^,^7^,^8^; indeed, it physically constrains the open seaway to a position north of the Ellsworth Mountains. There are two further implications. First, the agreement of our evidence with models driven by ocean temperature supports the long held view of the sensitivity of the WAIS to marine forcing. Second, our field results support numerical ice-sheet models that imply a WAIS contribution to global sea level of no more than ∼3.3 m above present^3^,^11^.

In conclusion, our results point to the continuous presence of an ice sheet in the southern Ellsworth Mountains sector of the WAIS for 1.4 Ma. The ice divide adjusted to accommodate the loss of marine-based portions of the main ice sheet during some interglacials, but a regional ice sheet based on the Ellsworth-Whitmore uplands was sufficient in size and altitude to maintain katabatic winds and form blue-ice moraines in the Heritage Range throughout the Pleistocene. Here, near the grounding line, the ice sheet has experienced a long-term trajectory of lowering relative to the mountains, one that is marked by modest fluctuations in thickness as it responded to glacial–interglacial climate and sea level cycles.

## Methods

### Geomorphology

Landforms were mapped in the field using differential global positioning satellite and satellite imagery. This formed the basis for detailed work on sediment morphology, lithology, weathering and cosmogenic isotope analysis. Selected areas, such as complex debris accumulations, were mapped with a laser scanner as well as high-resolution vertical aerial photographs taken from an unmanned aerial vehicle. The British Antarctic Survey Polarimetric-radar Airborne Science Instrument ice-sounding radar was used to image deeper englacial reflections (data collected during an airborne survey of the Institute Ice Stream, austral summer 2010/2011 (ref. 36)). A differential global positioning satellite system, mounted on a snowmobile, traversed along the ice margin of both massifs to provide a reference surface from which to normalise sample elevations. Topographic control on the ice margin introduces variability in elevation and therefore uncertainties in the normalization process are estimated at±15 m. Supplementary Fig. 1 shows the general ice flow configuration around the southern Heritage Range. Supplementary Fig. 2 shows a geomorphologic map of the Marble Hills massif. Supplementary Fig. 3 shows sample locations from the Patriot Hills. Supplementary Fig. 4 illustrates the nature of the glaciated upland surface and the type and distribution of weathered debris.

### Cosmogenic nuclide analysis

The cosmogenic nuclide data are presented in Supplementary Tables 1–3 and Supplementary Figs 5–6. All exposure ages discussed are based on ^10^Be ages because the production rate is better constrained; ^26^Al and ^21^Ne are used to constrain exposure histories. The measurement of ^21^Ne was completed because, as a stable isotope, it gives a measure of the total exposure time (assuming no erosion) irrespective of subsequent burial, and because it can potentially record longer periods of exposure than is generally possible with radioactive isotopes.

The sampling strategy for cosmogenic nuclides was designed to reduce the chance of nuclide inheritance, and exclude the possibility of nuclide loss through erosion. We targeted subglacially derived clasts with striated surfaces and subangular to subrounded shapes. We sampled the freshest appearing, quartz-bearing, brick-sized clasts resting on flat bedrock to minimize problems of post-depositional movement and self-shielding. It is crucial to be convinced that the exposure ages reflect the time since deposition of a freshly exposed clast rather than a signal inherited from the past. To test whether clasts emerging on the glacier surface in blue-ice areas have no inherited cosmogenic nuclides, we analysed seven clasts on the present ice margin. All had negligible amounts of both ^10^Be and ^26^Al, implying that they were first exposed to cosmic rays when they emerged at the ice-surface. In view of the similar lithologies, and thus origin, of quartz-rich erratics at higher elevations, it is reasonable to argue that they too were deposited with no significant pre-exposure. This is reinforced by the clustering of multi-isotope exposure ages from each sampled site. Thus, we conclude that the cosmogenic nuclide concentration in the erratics accurately reflects their exposure history since deposition.

### Laboratory and analytical techniques

Whole-rock samples were crushed and sieved to obtain the 250–710 μm fraction. Be and Al were selectively extracted from the quartz component of the whole-rock sample at the University of Edinburgh's Cosmogenic Nuclide Laboratory following established methods^37^,^38^. ^10^Be/^9^Be and ^26^Al/^27^Al ratios were measured in 20–30 g of quartz at the Scottish Universities Environmental Research Centre Accelerator Mass Spectrometry (AMS) Laboratory in East Kilbride, UK. Measurements are normalized to the NIST SRM-4325 Be standard material with a revised^39^ nominal ^10^Be/^9^Be of 2.79 × 10^−11^ and half-life of 1.387 Ma (refs 40, 41), and the Purdue Z92-0222 Al standard material with a nominal ^26^Al/^27^Al of 4.11 × 10^−11^, which agrees with the Al standard material of Nishiizumi^42^ with half-life of 0.705 Ma (ref. 43). Scottish Universities Environmental Research Centre ^10^Be-AMS is insensitive to ^10^B interference^44^ and the interferences to ^26^Al detection are well characterized^45^. Process blanks (*n*=6) were spiked with 250 μg ^9^Be carrier (Scharlau Be carrier, 1,000 mg l^−1^, density 1.02 g ml^−1^) and 1.5 mg ^27^Al carrier (Fischer Al carrier, 1,000 p.p.m.). Samples were spiked with 250 μg ^9^Be carrier and up to 1.5 mg ^27^Al carrier (the latter value varied depending on the native Al-content of the sample). Blanks range from 3.3 to 9.3 × 10^−15^ [^10^Be/^9^Be] (<1% of total ^10^Be atoms in all but the ice-margin samples); and 1.6–7.5 × 10^−15^ [^26^Al/^27^Al] (<1% of total ^26^Al atoms in all but the ice margin samples). Concentrations in Supplementary Table 1 are corrected for process blanks; uncertainties include propagated AMS sample/lab-blank uncertainty and a 2% carrier mass uncertainty and a 3% stable ^27^Al measurement inductively coupled plasma optical emission spectrometry uncertainty.

Neon isotopes were measured in ∼250 mg of leached quartz (250–500 μm). Samples were wrapped in aluminium foil and loaded into a Monax glass tree and evacuated to<10^−8^ torr for 48 h before analysis. Samples were successively heated for 20 min to 1,200 °C in a double-vacuum resistance furnace with a tungsten heating element and a molybdenum crucible. The extracted gas was cleaned on two hot SAES TiZr getters. The heavy noble gases (Ar, Kr and Xe) were absorbed onto a charcoal trap cooled with liquid nitrogen. Neon was then absorbed on to a charcoal trap at −228 °C for 20 min, and the residual He was removed by a turbomolecular pump. The Ne was released from the charcoal at −173 °C, and the isotopic composition analysed using a MAP 215–50 noble gas mass spectrometer. All Ne isotopes were measured in 11 peak jumping cycles using a Burle channeltron electron multiplier operated in pulse-counting mode. Neon abundances were determined by peak height comparison with Ne from 95.2±0.5 μcc STP air. The reproducibility of Ne abundances was better than ±1.5%, and isotopic ratios of replicate calibrations were better than ±0.5%. Interference corrections and detailed analytical procedure is presented elsewhere^46^. The ^20^Ne blank at 1,200 °C was typically ∼1 × 10^−11^ ccSTP and were indistinguishable from the atmospheric isotopic composition after correction for interfering species. Consequently no blank correction is made to the data in Supplementary Table 2. The consistency of procedures is demonstrated by the reproducibility of the cosmogenic ^21^Ne concentration in replicate analyses of MH12–27 (Supplementary Table 2). In all samples Ne isotope compositions are consistent with binary mixture of air and cosmogenic Ne. The ^21^Ne concentrations in Supplementary Table 2 include a correction for nucleogenic ^21^Ne (that is, non-cosmogenic ^21^Ne) of 7.7±2.4 × 10^6^ at g^−1^. This is the value estimated by Middleton *et al.*^47^ for Beacon Sandstone; we use this value as a best estimate based on the similar lithology and thermal history of the rocks. This value is close to the mode of the range of nucleogenic ^21^Ne measured in Antarctic rocks (see Balco and Shuster^48^ for a review). However, there is likely variability in the nucleogenic ^21^Ne concentrations that could impact the youngest samples. The conclusions are insensitive to uncertainty in burial time.

### Exposure age calculations

For exposure age calculations we used default settings in Version 2.0 of the CRONUScalc programme^49^. This is the product of the CRONUS-Earth collaboration that allows for all commonly used nuclides to be calculated using the same underlying framework, resulting in internally consistent cross-nuclide calculations for exposure ages, erosion rates and calibrations. The CRONUS-Earth production rates^50^ with the nuclide-dependent scaling of Lifton-Sato-Dunai^51^ were used to calculate the ages presented in the paper. Sea level and high latitude production rates are 3.92±0.31 atoms g^−1^ a^−1^ for ^10^Be and 28.5±3.1 atoms g^−1^ a^−1^ for ^26^Al. However, the use of Lal/Stone^26^,^52^ scaling does not change the conclusions of the paper despite the ∼3 and 8% older exposure ages for ^10^Be and ^26^Al, respectively. Rock density is 2.7 g cm^−3^ and the attenuation length used is 153±10 g cm^−2^. No corrections are made for rock surface erosion or snow cover and thus exposure ages are minima. Finally, we make no attempt to account for production rate variations caused by elevation changes associated with glacial isostatic adjustment of the massif through time^53^. This is justified because the samples have been exposed for multiple glacial cycles and thus any variations in elevation associated with ice loading and unloading, which has been of similar magnitude (maximum elevation difference 170 m), are likely to have been averaged out to the point of being smaller than other sources of uncertainty.

The CRONUScalc code for ^21^Ne was modelled after the existing code for ^3^He and only includes spallation production. The ^21^Ne production rate is tied to the total CRONUScalc ^10^Be production rate (assuming 1.5% production from muons^49^) with a ^21^Ne/^10^Be ratio of 4.08±0.37 (ref. 48), resulting in a ^21^Ne production rate of 16.26±1.96 atoms g^−1^ a^−1^ at sea level, high latitude scaled according to nuclide-dependent Lifton-Sato-Dunai^48^,^50^,^51^. There are several other alternative ^21^Ne production rates (all converted to be consistent with Lifton-Sato-Dunai scaling): 14.5 (Amidon *et al.*^54^, 18.0 (Vermeesch *et al.*^55^) and 18.9 (Niedermann *et al.*^56^). The Balco and Shuster^48^ rate was used because it is based on ratios tied to ^10^Be instead of ^26^Al, it uses a relatively large dataset compared with other ^21^Ne studies, it was performed using Antarctic samples, and the resulting rate falls in the middle of the production rate range. The differences in age using the other production rates given above range from 12% older to 14% younger than those given in the paper. While these changes are significant, the exposure ages are consistently similar or older than the corresponding ^10^Be and ^26^Al ages so the exact choice of ^21^Ne production rate does not affect the conclusions presented in the paper. For comparison, Lal/Stone^26^,^52^ scaling in CRONUScalc was used in conjunction with the production rate from Balco and Shuster^48^and produced ^21^Ne exposure ages that were approximately 3% younger than those produced using the nuclide-dependent Lifton-Sato-Dunai scaling scheme with the Balco and Shuster^48^ production rate.

Supplementary Figs 5 and 6 show plots of the isotopic ratios of ^26^Al/^10^Be and ^21^Ne/^10^Be. Samples should plot within the erosion island if they have been continuously exposed and eroding, and within the complex zone if they have been buried for a significant period of time, long enough for the shorter lived nuclide to preferentially decay. The ^26^Al/^10^Be system should be more sensitive to recent burial than the ^21^Ne/^10^Be system because of the shorter half-life of ^26^Al (0.705 ka). In our samples, the burial signal implied by the ^21^Ne/^10^Be ratios is greater than that implied by ^26^Al/^10^Be ratios. There are a few possible explanations. First, this may partly reflect the uncertainties on ^21^Ne production rates as discussed above. Second, it is possible that the samples contain additional nucleogenic ^21^Ne that has not been corrected for. A final explanation is that ^21^Ne, which is stable, is recording a period of exposure that is not evident in the ^26^Al/^10^Be system. At present it is not possible to discriminate between the above scenarios. In any case, our conclusions are not sensitive to these minor discrepancies.

### Till analysis for marine traces

Scherer (R. Scherer, personal communications, 2014) examined four till samples from both current and elevated blue-ice moraines and found no evidence of diatoms or biogenic silica.

## Additional information

**How to cite this article:** Hein, A. S*. et al.* Evidence for the stability of the West Antarctic Ice Sheet divide for 1.4 million years. *Nat. Commun.* 7:10325 doi: 10.1038/ncomms10325 (2016).

## Supplementary Material

Supplementary Information Supplementary Figures 1-6, Supplementary Tables 1-3 and Supplementary References
