# Supplementary material for: Evidence for the stability of the West Antarctic Ice Sheet divide for 1.4 million years
Source: Nat Commun. 2016 Feb 3;7:10325. doi: 10.1038/ncomms10325 (PMC4742792; doi:10.1038/ncomms10325)
Supplement: Supplementary Information — Supplementary Figures 1-6, Supplementary Tables 1-3 and Supplementary References [file ncomms10325-s1.pdf]

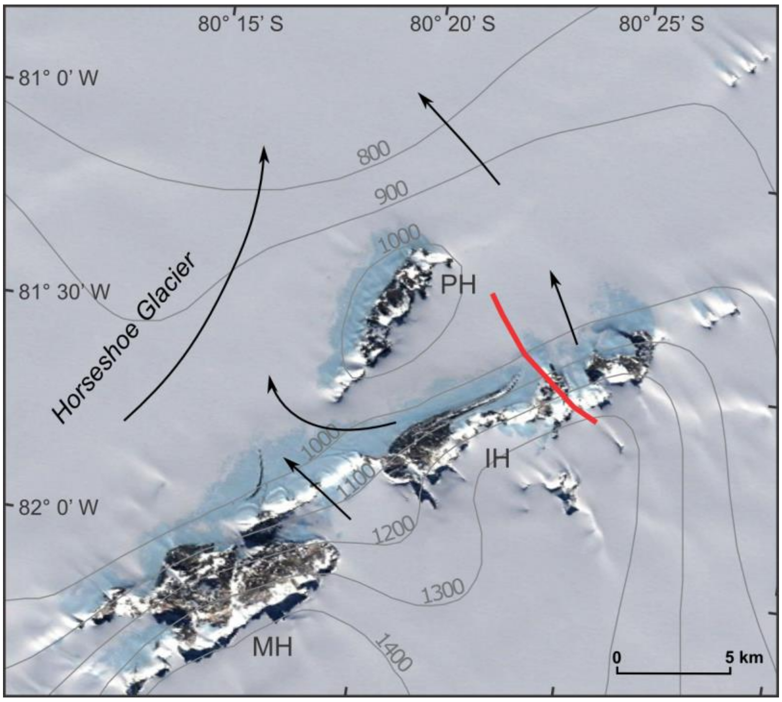

Supplementary Figure 1. The southern Heritage Range. The Google Earth imagery shows the location of the Patriot Hills (PH), Independence Hills (IH) and Marble Hills (MH) and current ice flow directions (black arrows). The red line indicates the location of the RES profile shown in Figure 2. Surface contours are from Bedmap2[1]

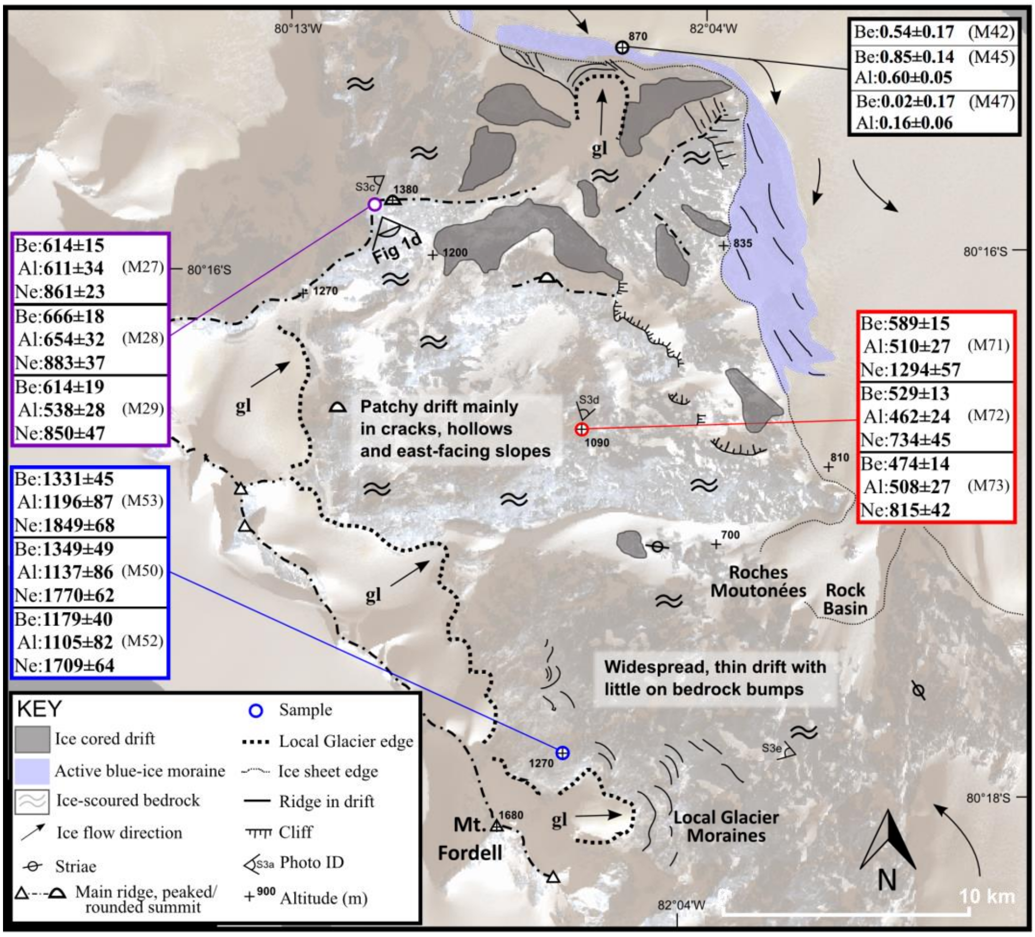

Supplementary Figure 2. The geomorphology of the Marble Hills. The figure shows current blue-ice moraines, ice-scoured uplands, elevated ice-cored tills, sampling sites of erratics and exposure ages (ka) and internal uncertainties (1 s.d.). The location and orientation of the photograph in Figure 1d of the main text is marked. The photo ID's beginning with "S" refers to panels in Supplementary Figure 4. The lithology of the quartz-rich erratics is similar at both the current and elevated margins. More easily weathered shales, slates and limestone are rare among the highest deposits. The colour-coding used is the same as in Supplementary Figures 5 and 6.

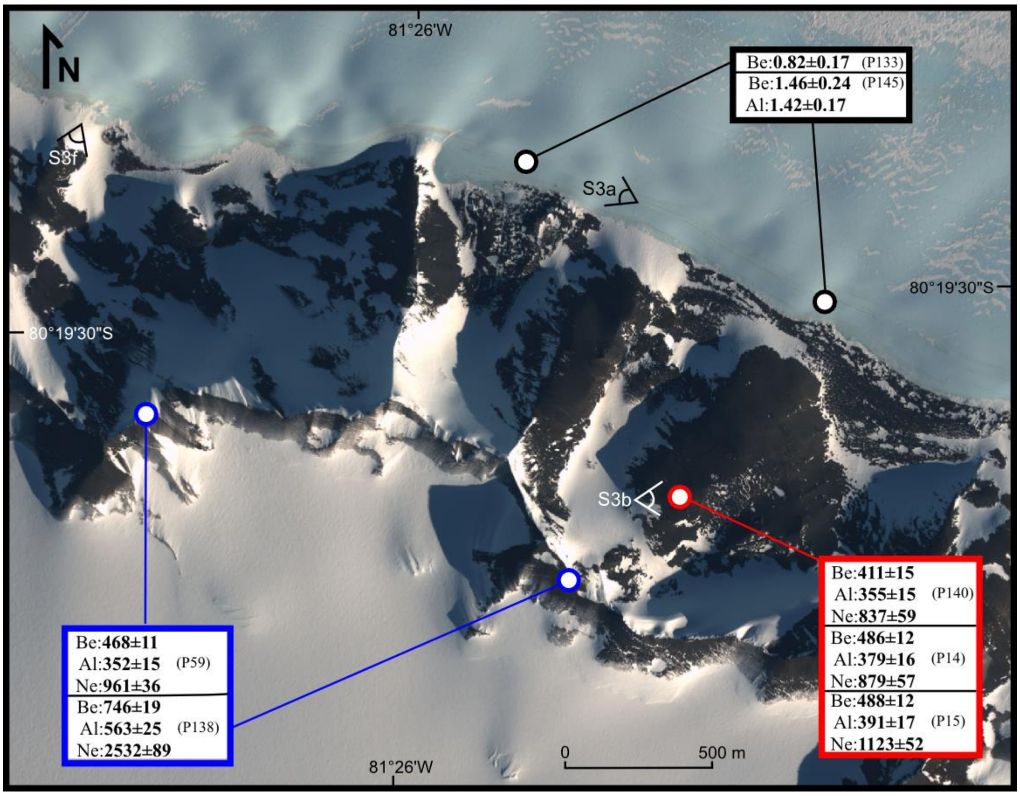

Supplementary Figure 3. Satellite Image of the Patriot Hills showing sample locations and exposure ages (ka) and internal uncertainties (1 s.d.). Two further samples were taken from the ice along the margin further east. The symbol and tag beginning with "S" refers to photos in the named panels in Supplementary Figure 4. The colour-coding used is the same as in Supplementary Figures 5 and 6.

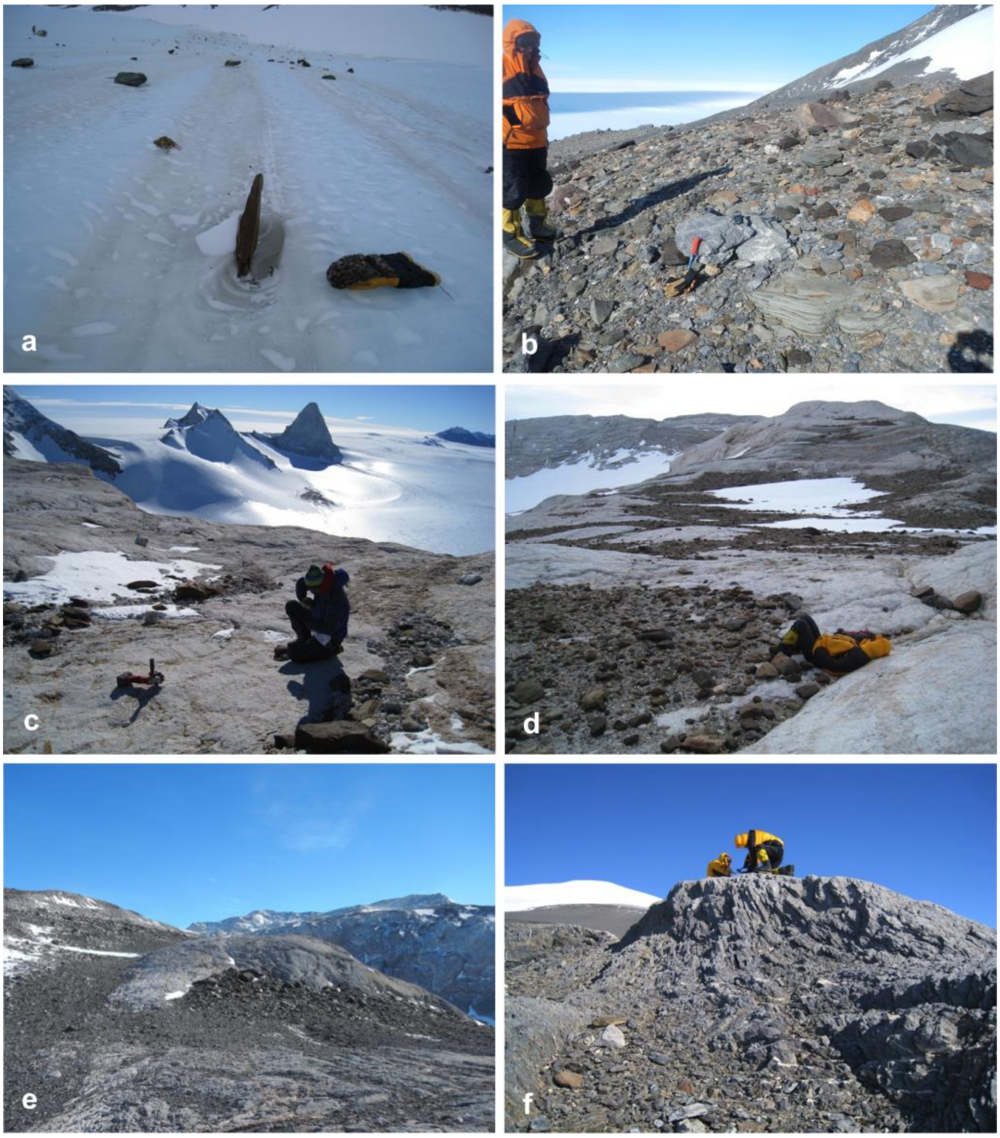

Supplementary Figure 4. Photos of glacial features. a) Debris emerging from folded debris bands in the blue-ice zone at the foot of the Patriot Hills, Heritage Range. The upward compressive ice flow compensates for ablation at the surface by strong katabatic winds and brings subglacial material to the surface. b) Elevated, weathered blue-ice till in the Patriot Hills. Seven ^{10}Be exposure dates here (four previously reported[2]) ranged from 400 – 500 ka with isotopic evidence of burial. c) Weathered, quartz-rich erratics lying on the summit of a dome of ice-scoured, limestone bedrock, Marble Hills, Heritage Range. Three erratics from this location had ^{10}Be exposure ages of 600-700 ka with little sign of burial subsequently. d) Patches of overridden till and erratics located in depressions and other east-facing topographic irregularities. Some trails of erratics are parallel to ice flow. Erratics here had ^{10}Be exposure ages of 500 - 600 ka with some isotopic ratios indicating burial. e) Overridden till and erratics deposited behind an east-facing topographic irregularity. f) A roche moutonnée, one of many such features with gentle west-facing slopes and plucked east-facing slopes on spurs on the northern flank of the Patriot Hills. This was likely formed during an early erosive glaciation. See Supplementary Figures 2 and 3 for the location of photographs.

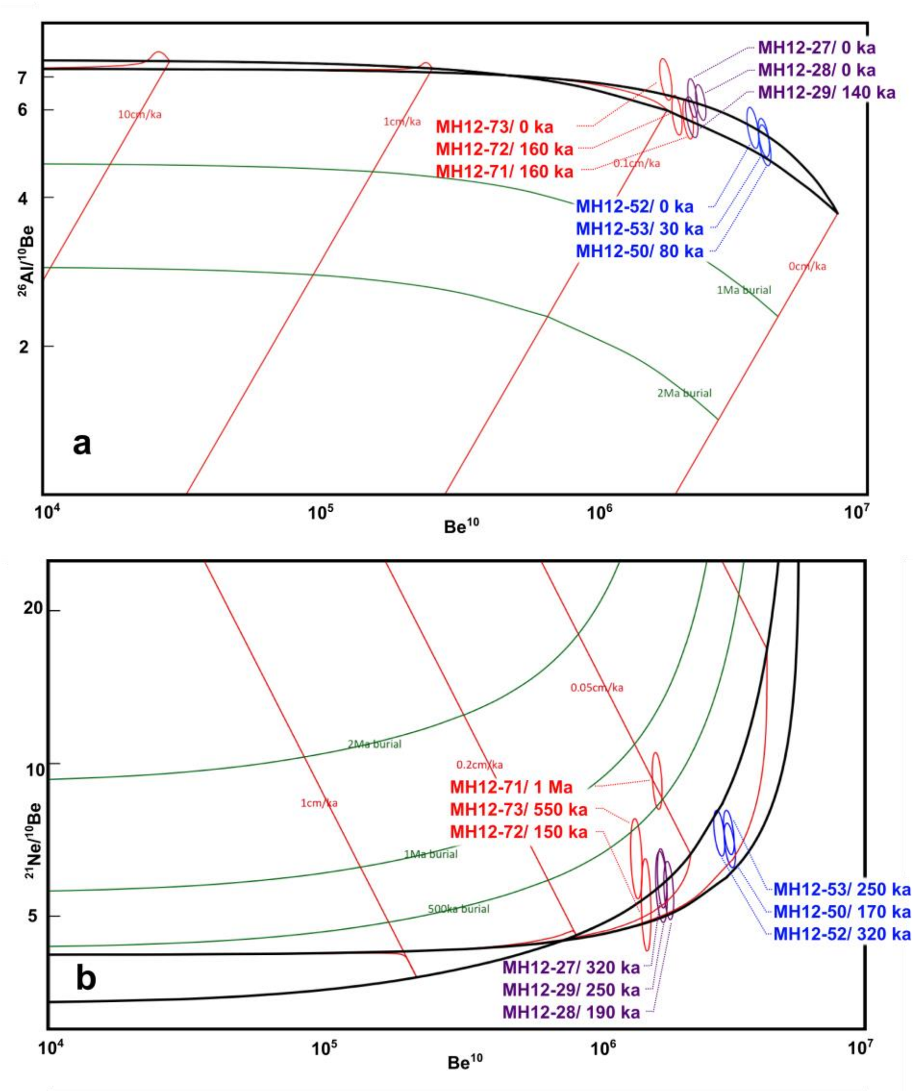

Supplementary Figure 5. Isotope ratio plots for Marble Hills. Samples in matching colours come from the same site within the upper weathered zone. In both plots, the area between the black lines is the “erosion island” which indicates ratios consistent with continuous surface exposure and erosion (i.e., no burial). However, assuming no erosion has taken place, the approximate *maximum* amount of burial (excluding uncertainties) is given for each sample. Note the burial ages assume zero erosion and thus differ from the green burial isochron, which is referenced to the erosion saturation line [lower line in (a); upper line in (b)]. a) $^{26}Al/^{10}Be$ plot; all samples have $^{26}Al/^{10}Be$ ratios that plot within the erosion island. (b) $^{21}Ne/^{10}Be$ plot; samples plotting above the upper black lines have a complex exposure involving periods of burial by ice. The Mt. Fordell samples (blue) and Marble Dome samples (purple) plot within the erosion island while MH12-71 and 72 indicate burial. The plots and burial ages were generated in the CosmoCalc programme[3] using production rates and scaling models described in the Methods section of the main text. Uncertainty ellipses are 1 s.d. See Supplementary Table 3 for exposure ages.

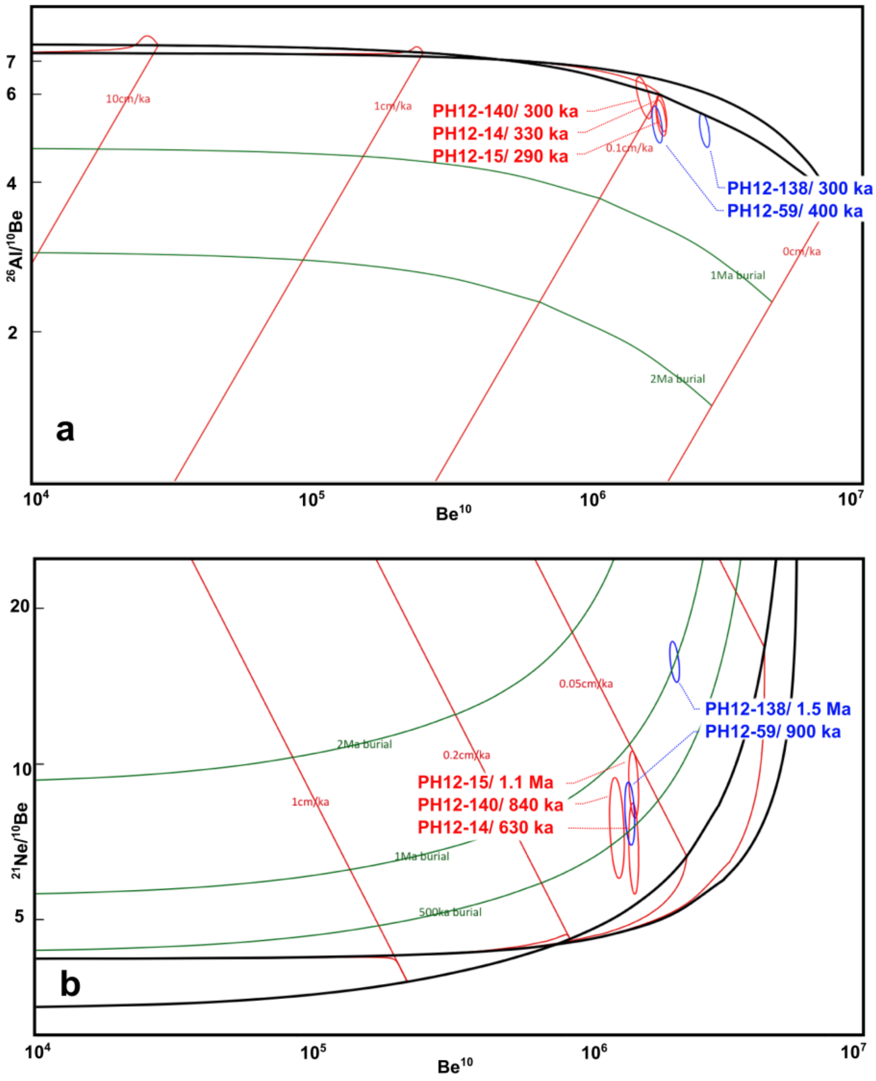

Supplementary Figure 6. Isotope ratio plots for Patriot Hills. Samples in matching colours come from the same or similar sites within the upper weathered zone. In both plots, the area between the black lines is the "erosion island" which indicates ratios consistent with continuous surface exposure and erosion (i.e., no burial). However, assuming no erosion has taken place, the approximate *maximum* amount of burial (excluding uncertainties) is given for each sample. Note the burial ages assume zero erosion and thus differ from the green burial isochron, which is referenced to the erosion saturation line [lower line in (a); upper line in (b)]. a) $^{26}Al/^{10}Be$ plot; samples plotting below the lower black lines have a complex exposure involving periods of burial by ice; all samples indicate burial. (b) $^{21}Ne/^{10}Be$ plot; samples plotting above the upper black lines have a complex exposure involving periods of burial by ice; all samples indicate burial. The burial ages and plots were generated in the CosmoCalc programme[3] using production rates and scaling models described in the Methods section of the main text. Uncertainty ellipses are 1 s.d. See Supplementary Table 3 for exposure ages.

Supplementary Table 1 **Rock sample details and cosmogenic ^{10}Be and ^{26}Al concentrations in quartz erratics.**

Sample ID	Lat. (dd)	Long. (dd)	Alt. (m asl)	Alt. above ice (m)	Lithology*	Thickness (cm)	Topo shielding	Quartz mass (g)	^{10}Be AMS ID†	^{10}Be concentration§ ± 1σ (10^5 atom g^{-1}[SiO_2])	^{26}Al AMS ID†	^{26}Al concentration# ± 1σ (10^5 atom g^{-1}[SiO_2])
MH12-53	-80.2967	-82.1180	1271	649	Qtzite	4.5	0.988	31.7907	b8393	155.889 ± 3.708	a2149	811.594 ± 30.764
MH12-52	-80.2968	-82.1170	1269	647	Qtz	3	0.988	27.2575	b8392	144.457 ± 3.565	a2148	786.496 ± 32.174
MH12-50	-80.2966	-82.1150	1267	645	SS	5	0.989	26.5943	b8391	156.295 ± 3.933	a2147	784.680 ± 31.825
MH12-27	-80.2631	-82.1871	1381	551	SS	3	1.000	29.0853	b7350	95.885 ± 1.982	a1850	598.927 ± 23.883
MH12-28	-80.2626	-82.1863	1380	550	SS	2.5	1.000	11.0093	b7351	103.067 ± 2.281	a1851	630.531 ± 22.119
MH12-29	-80.2625	-82.1863	1375	545	SS	5	1.000	22.3397	b8387	93.729 ± 2.433	a2146	531.953 ± 21.166
MH12-72	-80.2766	-82.1105	1095	316	SS	4.5	0.998	23.5256	b8403	64.198 ± 1.384	a2155	369.864 ± 15.167
MH12-71	-80.2766	-82.1101	1093	314	SS	3.5	0.998	25.8063	b8400	71.001 ± 1.545	a2154	402.161 ± 16.459
MH12-73	-80.2766	-82.1106	1092	313	Qtzite	5	0.998	25.6635	b8404	57.904 ± 1.473	a2156	395.855 ± 16.346
MH12-45	-80.2529	-82.0915	879	0	SS	4	0.999	26.5383	b7355	0.102 ± 0.020	a1852	0.518 ± 0.075
MH12-47	-80.2533	-82.0868	869	0	SS	3.5	0.998	18.7105	b7356	0.002 ± 0.017	a1853	0.126 ± 0.066
MH12-42	-80.2529	-82.1038	867	0	SS	6	0.998	22.5694	b8390	0.062 ± 0.020	na	na
PH12-138	-80.3337	-81.4099	1189	344	Red SS	6	0.998	24.5112	b8380	92.610 ± 1.952	a2139	463.256 ± 15.631
PH12-59	-80.3275	-81.4821	1109	264	Red SS	6	0.999	25.3822	b7333	57.793 ± 1.243	a1833	296.636 ± 10.913
PH12-140	-80.3308	-81.3862	1006	271	Red SS	2	0.991	25.3823	b8381	48.097 ± 1.544	a2140	279.486 ± 10.028
PH12-14	-80.3308	-81.3864	997	262	Red SS	3.0	0.990	29.022	b7327	54.750 ± 1.176	a1826	290.042 ± 10.362
PH12-15	-80.3309	-81.3850	997	262	Red SS	5	0.990	28.4429	b7328	54.033 ± 1.166	a1827	292.573 ± 10.487
PH12-133	-80.3200	-81.4135	762	0	SS	3	0.995	25.6513	b8379	0.089 ± 0.015	na	na
PH12-145	-80.3252	-81.3540	751	0	SS	5	0.996	25.9485	b7337	0.152 ± 0.023	a1838	1.074 ± 0.106
PH12-96	-80.3300	-81.2939	742	0	SS	3.5	0.997	27.098	b7334	0.135 ± 0.022	a1834	0.678 ± 0.083
PH12-97	-80.3303	-81.2901	738	0	Qtz SS	3	0.997	23.0313	b8371	0.008 ± 0.012	na	na

*SS: sandstone; Qtz: quartz; Qtzite: quartzite. Rock density 2.7 g cm^{-3}; attenuation length 153 ± 10 g cm^2.

†AMS measurements made at Scottish Universities Environmental Research Centre (SUERC).

§Normalised to NIST SRM-4325 Be standard material with a revised nominal $^{10}Be/^{9}Be$ ratio (2.79 x 10^{-11})[4] and half-life of 1.387 Ma[5,6] and corrected for process blanks; uncertainties include propagated AMS sample/lab-blank uncertainty and a 2% carrier mass uncertainty.

#Normalised to the Purdue Z92-0222 Al standard material with a nominal $^{27}Al/^{26}Al$ ratio of 4.11 x 10^{-11} that agrees with Al standard material of (7), and corrected for process blanks; uncertainties include propagated AMS sample/lab-blank uncertainty and a 3% stable ^{27}Al measurement (ICP-OES) uncertainty.

Supplementary Table 2. Cosmogenic Ne data from quartz erratics.

Sample ID	$^{21}Ne/^{20}Ne$	± 1σ	$^{22}Ne/^{20}Ne$	± 1σ	$^{21}Ne_{cos}$ (10^6 atom g^{-1} [SiO_2])#	± 1σ
MH12-53	0.0049	3.81E-05	0.103	3.46E-04	114.50	5.07
MH12-52	0.0044	2.97E-05	0.104	3.95E-04	105.92	4.87
MH12-50	0.0074	7.90E-05	0.107	5.17E-04	108.09	4.73
MH12-27 (a)	0.0068	5.37E-05	0.106	4.75E-04	55.84	3.21
MH12-27 (b)	0.0064	9.97E-05	0.105	6.40E-04	57.91	3.71
MH12-28	0.0043	3.72E-05	0.103	3.81E-04	58.37	3.64
MH12-29	0.0043	6.04E-05	0.103	3.80E-04	54.26	4.16
MH12-72	0.0035	2.93E-05	0.103	3.18E-04	34.05	3.52
MH12-71	0.0038	2.64E-05	0.103	4.35E-04	66.33	4.04
MH12-73	0.0040	3.91E-05	0.103	3.68E-04	38.25	3.36
PH12-138	0.0048	3.23E-05	0.104	3.76E-04	146.91	5.93
PH12-59	0.0062	6.60E-05	0.105	6.79E-04	47.01	3.15
PH12-140	0.0036	3.78E-05	0.102	3.59E-04	36.79	3.97
PH12-14	0.0037	3.92E-05	0.102	3.49E-04	38.17	3.8
PH12-15	0.0055	8.32E-05	0.105	5.15E-04	49.95	3.58

MS measurements conducted at SUERC.

#Includes correction for nucleogenic ^{21}Ne of 7.7 ± 2.4 x 10^6 at g^{-1} [(8)].

Supplementary Table 3. **Cosmogenic ^{10}Be, ^{26}Al and ^{21}Ne surface exposure ages.**

Sample ID	Alt. above ice (m)	^{10}Be age* ± 1σ (int)† (ka)	± 1σ (ext)† ^{10}Be (ka)	± 1σ (full)† ^{10}Be (ka)	^{26}Al age* ± 1σ (int)† (ka)	± 1σ (ext)† ^{26}Al (ka)	± 1σ (full)† ^{26}Al (ka)	^{21}Ne age* ± 1σ (int)† (ka)	± 1σ (ext)† ^{21}Ne (ka)	± 1σ (full)† ^{21}Ne (ka)	^{26}Al/^{10}Be ± 1σ	^{21}Ne/^{10}Be ± 1σ
MH12-53	649	1331 ± 45	153	220	1196 ± 87	255	323	1849 ± 68	231	275	5.21 ± 0.23	7.84 ± 0.34
MH12-52	647	1179 ± 40	131	187	1105 ± 82	226	284	1709 ± 64	214	255	5.44 ± 0.26	7.87 ± 0.35
MH12-50	645	1349 ± 49	157	225	1137 ± 86	237	297	1770 ± 62	220	263	5.02 ± 0.24	7.41 ± 0.32
MH12-27#	551	614 ± 15	58	82	611 ± 34	95	117	861 ± 23	77	91	6.25 ± 0.28	6.73 ± 0.29
MH12-28	550	666 ± 18	64	91	654 ± 32	103	127	883 ± 37	112	133	6.12 ± 0.25	6.41 ± 0.30
MH12-29	545	614 ± 19	59	83	538 ± 28	80	99	850 ± 47	112	131	5.68 ± 0.27	6.61 ± 0.40
MH12-72	316	529 ± 13	49	69	462 ± 24	67	82	734 ± 45	99	115	5.76 ± 0.27	6.50 ± 0.43
MH12-71	314	589 ± 15	55	79	510 ± 27	75	93	1294 ± 57	165	195	5.66 ± 0.26	10.43 ± 0.51
MH12-73	313	474 ± 14	44	62	508 ± 27	75	93	815 ± 42	106	125	6.84 ± 0.33	7.94 ± 0.45
MH12-45	0	0.85 ± 0.14	0.16	0.18	0.60 ± 0.05	0.08	0.09				5.08 ± 1.24	
MH12-47	0	0.02 ± 0.17	0.17	0.17	0.16 ± 0.06	0.06	0.07				62.11 ± 506.94	
MH12-42	0	0.54 ± 0.20	0.21	0.21								
PH12-138	344	746 ± 19	73	104	563 ± 25	84	104	2532 ± 89	315	376	5.00 ± 0.20	16.70 ± 0.68
PH12-59	264	468 ± 11	42	61	352 ± 15	47	58	961 ± 36	120	143	5.13 ± 0.22	9.47 ± 0.41
PH12-140	271	411 ± 15	38	54	355 ± 15	48	59	837 ± 59	116	135	5.81 ± 0.28	9.25 ± 0.72
PH12-14	262	486 ± 12	44	63	379 ± 16	51	64	879 ± 57	119	139	5.30 ± 0.22	8.38 ± 0.57
PH12-15	262	488 ± 12	44	64	391 ± 17	53	66	1123 ± 52	144	170	5.41 ± 0.23	10.67 ± 0.54
PH12-133	0	0.82 ± 0.17	0.18	0.2								
PH12-145	0	1.46 ± 0.24	0.26	0.29	1.42 ± 0.17	0.20	0.24				7.04 ± 1.29	
PH12-96	0	1.28 ± 0.26	0.29	0.31	0.89 ± 0.14	0.19	0.20				5.03 ± 1.04	
PH12-97	0	0.09 ± 0.16	0.16	0.16								

*Ages calculated with CRONUS-Earth CRONUScalc v.2.0[9], LSD scaling[10], no erosion correction.

^{26}Al and ^{10}Be production rates from CRONUS-Earth Project[11]; The ^{21}Ne production rate is tied to the total CRONUScalc ^{10}Be production rate (assuming 1.5% production from muons[9]) with a ^{21}Ne/^{10}Be ratio of 4.08 ± 0.37[12].

#Average of two replicate ^{21}Ne concentrations used in the age determination for this sample.

†(int) = Internal uncertainties; includes only concentration uncertainties based on lab/AMS measurements.

†(ext) = External uncertainties; includes internal uncertainties plus scaling and production rate uncertainties.

†(full) = Full uncertainties; includes external uncertainties plus uncertainties on thickness (0.5 cm), pressure (10hPa), attenuation length (10g/cm^2), and density (0.05 g/cm^3).

Supplementary References

1 Fretwell, P. *et al.* Bedmap2: improved ice bed, surface and thickness datasets for Antarctica. *Cryosphere* **7**, 375-393, doi:10.5194/tc-7-375-2013 (2013).

2 Fogwill, C. J., Hein, A. S., Bentley, M. J. & Sugden, D. E. Do blue-ice moraines in the Heritage Range show the West Antarctic ice sheet survived the last interglacial? *Palaeogeography Palaeoclimatology Palaeoecology* **335**, 61-70, doi:10.1016/j.palaeo.2011.01.027 (2012).

3 Vermeesch, P. CosmoCalc: An Excel add-in for cosmogenic nuclide calculations. *Geochemistry Geophysics Geosystems* **8**, doi:10.1029/2006GC001530 (2007).

4 Nishiizumi, K. *et al.* Absolute calibration of Be-10 AMS standards. *Nuclear Instruments & Methods in Physics Research Section B* **258**, 403-413, doi:10.1016/j.nimb.2007.01.297 (2007).

5 Chmeleff, J., von Blanckenburg, F., Kossert, K. & Jakob, D. Determination of the Be-10 half-life by multicollector ICP-MS and liquid scintillation counting. *Nuclear Instruments & Methods in Physics Research Section B-Beam Interactions with Materials and Atoms* **268**, 192-199, doi:10.1016/j.nimb.2009.09.012 (2010).

6 Korschinek, G. *et al.* A new value for the half-life of Be-10 by Heavy-Ion Elastic Recoil Detection and liquid scintillation counting. *Nuclear Instruments & Methods in Physics Research Section B-Beam Interactions with Materials and Atoms* **268**, 187-191, doi:10.1016/j.nimb.2009.09.020 (2010).

7 Nishiizumi, K. Preparation of Al-26 AMS standards. *Nuclear Instruments & Methods in Physics Research Section B* **223-24**, 388-392, doi:10.1016/j.nimb.2004.04.075 (2004).

8 Middleton, J. L., Ackert, R. P., Jr. & Mukhopadhyay, S. Pothole and channel system formation in the McMurdo Dry Valleys of Antarctica: New insights from cosmogenic nuclides. *Earth Planet. Sci. Lett.* **355**, 341-350, doi:10.1016/j.epsl.2012.08.017 (2012).

9 Marrero, S. *et al.* Cosmogenic Nuclide Systematics and the CRONUScalc Program. *Quaternary Geochronology* CRONUS-Earth Special Volume, doi:10.1016/j.quageo.2015.09.005 (2015).

10 Lifton, N., Sato, T. & Dunai, T. J. Scaling in situ cosmogenic nuclide production rates using analytical approximations to atmospheric cosmic-ray fluxes. *Earth Planet. Sci. Lett.* **386**, 149-160, doi:10.1016/j.epsl.2013.10.052 (2014).

11 Borchers, B. *et al.* Geological calibration of spallation production rates in the CRONUS-Earth project. *Quaternary Geochronology*, doi:10.1016/j.quageo.2015.01.009.

12 Balco, G. & Shuster, D. L. Production rate of cosmogenic Ne-21 in quartz estimated from Be-10, Al-26, and Ne-21 concentrations in slowly eroding Antarctic bedrock surfaces. *Earth Planet. Sci. Lett.* **281**, 48-58, doi:10.1016/j.epsl.2009.02.006 (2009).
